# Chronic Chagas Cardiomyopathy in the Brazilian Amazon region: clinical characteristics and regional distinctiveness

**DOI:** 10.3389/fpubh.2023.1284639

**Published:** 2023-11-27

**Authors:** Elsa Isela Guevara Moctezuma, Susan Smith Doria, Jessica Vanina Ortiz, Débora Raysa Teixeira de Sousa, Victor Irungu Mwangi, Katia do Nascimento Couceiro, Alba Regina Jorge Brandão, Jorge Augusto de Oliveira Guerra, Maria das Graças Vale Barbosa Guerra, João Marcos Barbosa Bemfica Ferreira

**Affiliations:** ^1^Programa de Pós-Graduação em Medicina Tropical, Universidade do Estado do Amazonas, Manaus, Brazil; ^2^Gerência de Leishmaniose e Gerencia de Entomologia, Fundação de Medicina Tropical Dr. Heitor Vieira Dourado, Manaus, Brazil

**Keywords:** Chagas disease, chronic chagasic cardiopathy, Brazilian Amazon, heart failure, LBBB, left bundle branch block

## Abstract

**Objectives:**

This study aims to provide a comprehensive analysis of clinical and epidemiological data related to Chronic Chagas Cardiomyopathy (CCC) in the Amazon region of Brazil.

**Methods:**

A review of observational, retrospective, and cross-sectional studies related to Chagas Disease in the Amazon region of Brazil was conducted, and a case series addressing CCC in patients treated at the FMT-HVD outpatient clinic, a reference center for Chagas disease in Brazil, was carried out.

**Results:**

Clinical characteristics of 55 patients from the Amazon region with CCC were described. The most common electrocardiographic alteration observed was abnormal ventricular repolarization (AVR), present in 40% of cases. The most common echocardiographic finding was left ventricular systolic dysfunction (49%), followed by akinesia or hypokinesia of the inferior and/or inferolateral walls (38.1%) and the presence of an apical aneurysm (32.7%).

**Conclusions:**

Overall, this study demonstrates that CCC in the Amazon region presents clinical characteristics and severity that are similar to those observed in other regions. However, certain peculiarities, such as the frequency of right bundle branch block (RBBB) and anterior and septal involvement during the acute phase, require additional investigation to better comprehend the disease in the region. Overall, the study provides crucial clinical insights for the diagnosis and treatment of CCC in the Amazon region.

## Introduction

Chagas disease (CD) is a significant global public health concern that is endemic to South America and is now emerging in Europe and the United States (1). It is estimated that there are between 6 to 7 million carriers of the disease worldwide, making it a significant health issue (2).

Chronic Chagas Cardiomyopathy (CCC) is caused by *Trypanosoma cruzi (T. cruzi)*, a versatile protozoan that utilizes different vertebrate (mammals) and invertebrate (hematophagous insects of the order Hemiptera) hosts during its life cycle, ultimately reaching humans through various transmission routes. (3). In 2009, a classification of *T. cruzi* was proposed based on six Discrete Typing Units (DTUs) (TcI to TcVI), which are differentially distributed among vertebrate and invertebrate hosts in different geographical areas of the American continent (4).

CD can manifest in two phases: an acute phase, which may be asymptomatic or symptomatic, and a chronic phase, which includes the indeterminate, cardiac, digestive, neurological or mixed forms. In traditionally endemic areas, approximately 60% of individuals are carriers of the indeterminate form, 30% are at risk of developing cardiac alterations, and up to 10% may present with digestive, neurological, or mixed alterations (3).

In the context of reactivation, the neurological form of CD can be particularly concerning. This reactivation predominantly occurs in immunocompromised individuals and can entail severe neurological implications affecting both the central nervous system (CNS) and peripheral nervous system. The reactivated infection may precipitate serious and potentially life-threatening complications, including meningoencephalitis (inflammation of brain and surrounding tissues) and neuropathy, characterized by symptoms like numbness, tingling, muscle weakness, and atrophy, alongside other neurological disorders (5, 6).

The chronic phase of Chagas disease can cause significant morbidity and mortality, particularly due to its cardiac involvement, which is the leading cause of non-ischemic cardiomyopathy in Latin America (7).

The classical pathological features of CCC include low-grade myocarditis accompanied by myocytolysis, hypertrophy of myofibers, and interstitial fibrosis. The scarring and fibrosis related to the chronic phase are prominent features that can be focal or diffuse, involving almost any area of the myocardium (8).

The symptoms and physical signs present in CCC stem from four essential syndromes that commonly coexist in the same patient: heart failure, arrhythmias, thromboembolism, and angina manifestations. These syndromes may cause a variety of clinical manifestations, including shortness of breath, chest pain, palpitations, edema, and fatigue. Additionally, they encompass dizziness, fainting, and syncope, as well as abdominal discomfort and loss of appetite (9). The prognosis of CCC varies based on disease severity, patient health, and treatment response. Adherence to prescribed regimens and access to specialized healthcare are vital for improving CCC patients' quality of life and outcomes. Individuals diagnosed with CCC require consistent monitoring and management by a cardiologist. This involves periodic medical history reviews, thorough physical examinations, and essential diagnostic tests (chest radiography, EKG, 24-hour Holter monitoring, echocardiogram) (7). These evaluations inform tailored treatment plans, including medication adjustments and lifestyle recommendations, to optimize cardiac health and improve the patient's quality of life. Cardiological follow-up is crucial for disease progression tracking and effective intervention, ultimately enhancing prognosis and overall cardiac health (7).

In the Amazon region, the prevalence of chronic CCC appears notably diminished compared to other regions in Brazil. Data from the Ministry of Health's 2022 bulletin indicate significantly lower vulnerability indices for chronic Chagas disease in specific states: Amapá (0.115), Amazonas (0.123), and Rio Grande do Norte (0.129). These indices were meticulously calculated based on a comprehensive array of parameters, including self-reported Chagas disease registrations, Chagas disease mortality, hospital admissions characterized by CID-10 coding for Chagas disease, CID-10 sentinel mortality for the cardiac form, hospitalizations due to heart failure, population coverage, percentage of heart failure admissions featuring Chagas disease serology, and the ratio of outpatient procedures pertaining to Chagas disease (10). Several hypotheses have been proposed to explain the better evolution and life expectancy of individuals with CCC in the Amazon region. The most studied hypothesis is the relationship between the pathogenicity and genetic lineage (DTU) of the parasite. In Central and North America, the DTU TcI has been reported as predominant and the main cause of CD in both acute and chronic phases (11, 12).

In 2012 a study, analyzed 96 samples of *T. cruzi* from populations in four municipalities in the western Amazon region and demonstrated that TcI and TcIV were the main DTUs found, with a predominance of TcIV among human cases of two outbreaks of acute CD through oral transmission (13).

Patients with chronic CD typically have low parasitemia, making it difficult to isolate and identify the circulating *T. cruzi* lineage. However, in 2013, a study detected DTU TcI in triatomines used for the xenodiagnosis of 13 out of 36 patients from the rural area of Manaus, in the state of Amazonas, Brazil. This finding suggests that TcI and TcIV are the main DTUs present in the Amazon region, with TcIV being the predominant lineage among human cases of acute CD caused by oral transmission. The lower prevalence and milder clinical features of CD in the Amazon region may be related to differences in the pathogenicity of *T. cruzi* lineages present in the area (14).

The prevalence and characteristics of CCC in the Amazon region remain significantly underexplored and the available data on this ailment in this specific geographic area is quite restricted. Furthermore, the distinctive attributes of CD within the Amazon region, which encompass unique clinical and epidemiological features, may give rise to distinct clinical presentations compared to individuals with CCC from other endemic regions. These unique attributes could include variations in the genetic lineage (DTU) of the parasite, demographic factors, differences in disease progression rates, as well as potential variations in the clinical manifestations and severity of the disease. As a result, the primary aim of this article is to provide comprehensive clinical and epidemiological data concerning CCC in the Brazilian Amazon, shedding light on these distinctive features for a better understanding and management of the disease in this region.

## Methods

This article presents two approaches with data on CCC:

1. A comprehensive review of observational, retrospective, and cross-sectional studies related to Chagas Disease (Chagas Disease) in the Brazilian Amazon region was conducted. This literature review included all available observational studies, cohort studies and case reports published in both English and Portuguese, with no limitations on the publication date.
2. A case series on CCC in the Brazilian Amazon, involving patients treated at the outpatient clinic of the Fundação de Medicina Tropical Dr. Heitor Vieira Dourado (FMT-HVD), a reference center for CD in the state of Amazonas.

### Sources of information and literature search for the review

Searches were systematically performed in several reputable databases, namely PUBMED, EMBASE, SCIELO, and Google Scholar. The search strategy incorporated a set of predefined keywords: “Chagas cardiomyopathy,” “heart failure,” “Chagas,” and “Amazon.” Boolean operators, including “AND” and “OR,” were employed to enhance the search precision. Specifically, the Boolean operator “OR” was utilized within groups of synonyms or related terms (e.g., “Chagas cardiomyopathy” OR “heart failure” OR “Chagas”) to broaden the scope of the search and capture a comprehensive range of relevant articles. Conversely, the operator “AND” was employed to refine the search, ensuring that the retrieved articles contained a combination of keywords [e.g., (“Chagas cardiomyopathy” OR “heart failure” OR “Chagas”) AND “Amazon”]. Additionally, we optimized the sensitivity of our information retrieval by incorporating free text in article titles and abstracts. Specifically, we utilized the precise phrase 'Chagas cardiomyopathy in the Amazon' as the primary search query. Employing Google Scholar as our database of choice, we further refined our search by applying the relevance filter, prioritizing the most significant results. Our focus was on identifying the most pertinent and informative sources within the first 50 results retrieved using this approach. This search was executed in December 2022, ensuring the inclusion of the most recent and up-to-date information available at that time.

### Case series

The study population consisted of patients from the Amazon region who were diagnosed with CCC between 2007 and 2022. The diagnosis was confirmed by at least two reactive serology tests based on different principles or with different antigenic preparations, such as ELISA, IFI, HAI, WB, or CLIA. In addition, the patients presented electrocardiogram and/or echocardiographic abnormalities suggestive of CCC. The clinical and epidemiological data were collected directly during patients' appointments and/or from their medical records, which are stored in the hospital's iDoctor digitalized medical records system. iDoctor is an electronic system that centralizes and organizes patients' medical information in a digital format. This approach enhances accessibility, updating, and efficient handling of clinical data, playing a fundamental role in the accurate collection of information for both clinical and epidemiological purposes.

### Cardiology studies

During the assessment, patients underwent clinical examination for evaluation of cardiac symptoms and general physical examination. In addition, all patients underwent additional cardiological examinations, such as electrocardiogram (ECG), echocardiogram, and 24-h holter monitoring, for evaluation of cardiac involvement and degree of ventricular dysfunction.

a) Electrocardiogram targeting the electrocardiographic alterations attributed to Chagas disease, such as: right bundle branch block (RBBB), Anterosuperior left divisional block (ASLBB), Left bundle branch block (LBBB), Atrioventricular block (AVB), Sinus bradycardia, Atrial fibrillation (AF), Ventricular arrhythmias, pathological Q waves or loss of R wave progression from V1 to V3-V4, and alteration of ventricular repolarization (AVR) (7).
b) 24-h Holter monitoring was performed to analyze the occurrence of alterations such as ventricular premature beats (VPBs), supraventricular premature beats (SVPBs), non-sustained supraventricular tachycardia (NSST), and paroxysmal supraventricular tachycardia (PSVT).
c) Ecocardiogram, abnormal findings include: left ventricular ejection fraction (LVEF) < 52% in men or 54% in women, dilated chambers (left ventricular end diastolic diameter > 56 mm), indexed left atrial volume >34 mL/m^2^, segmental myocardial contractility abnormalities, presence of apical aneurysm or moderate-to-severe valvular involvement.

For the stages of CCC evolution, criteria were followed according to Table 1, which categorizes five different groups (7).

**Table 1:** Initial staging of myocardial involvement in CCC.

**Stages**	***ECG***	**Echocardiogram**	**Heart failure**
A	Abnormal	Normal	Absent
B1	Abnormal	Abnormal, LVEF ≥ 45%	Absent
B2	Abnormal	Abnormal, LVEF < 45%	Absent
C	Abnormal	Abnormal	Compensable
D	Abnormal	Abnormal	Refractory

ECG, electrocardiogram; LVEF, left ventricular ejection fraction.

### Data analysis

Statistical analyses were performed using Stata/MP 14.1 software. In the descriptive study, continuous variables were represented as mean ± standard deviation (SD). For qualitative variables, frequencies and percentages were calculated. Furthermore, alternative measures of central distribution, including the median and the interquartile range (IQR), were presented to provide a more robust perspective.

### Ethical considerations

The FMT-HVD's Research Ethics Committee approved this study under the CAAE number 47017121.6.0000.0005.

## Results

### Literature review

**Figure 1:**
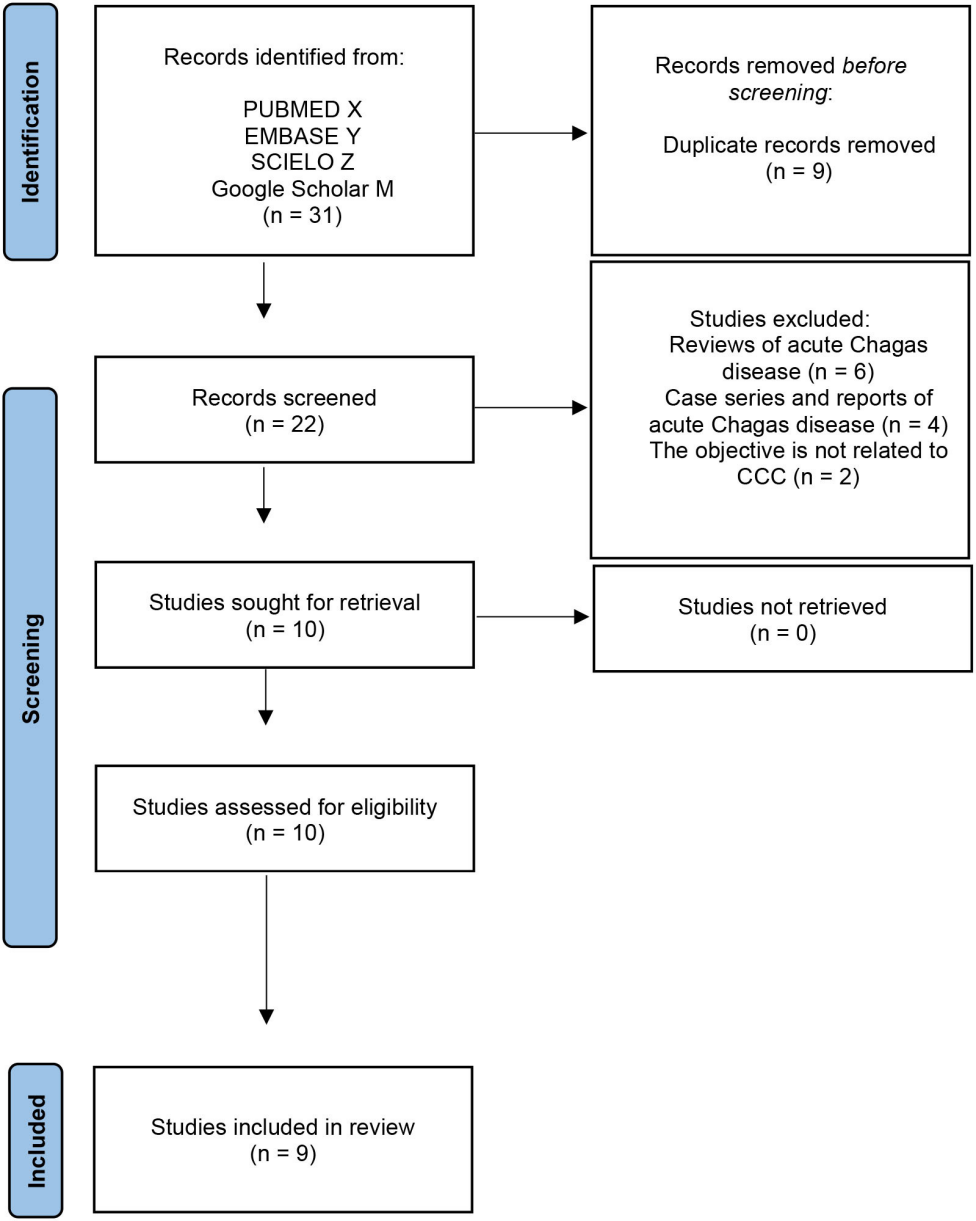
Overview of the screening process.

**Figure 2:**
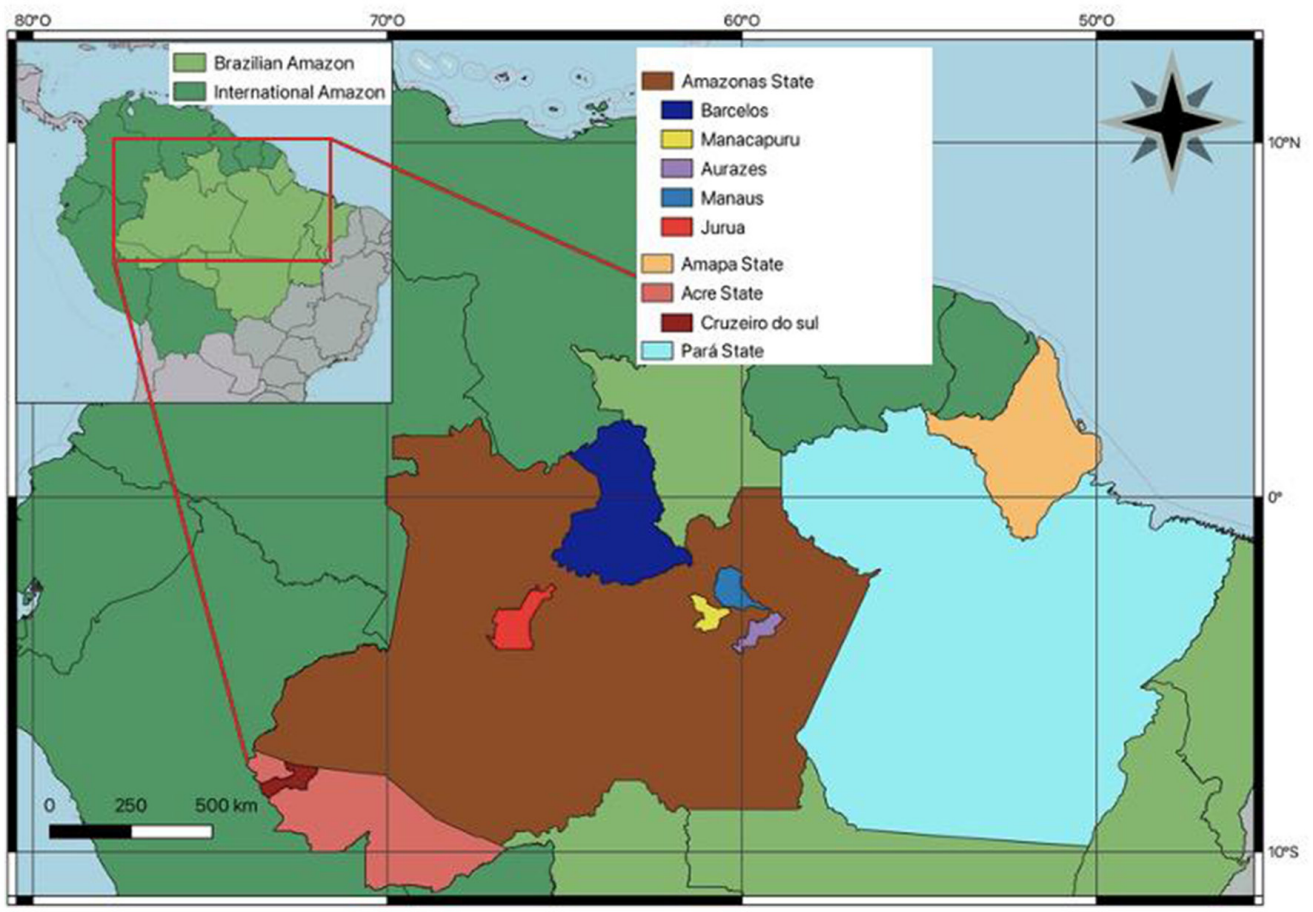
The regions in the Brazilian Amazon where CCC has been studied and identified, along with the cases discussed in this review, are marked on the map.

Using the selected keywords, a total of 22 articles related to chronic Chagas disease were identified. Subsequently, a comprehensive review of these articles was conducted, resulting in the exclusion of 12 of them. Of these, six were general reviews on acute Chagas disease, four were case series or individual case reports of acute Chagas disease, and two did not specifically address CCC, as detailed in Figure 1. These exclusion criteria were applied as part of our approach to assess article quality and prevent bias in the selection. The quality evaluation of the selected articles was carried out by applying predefined criteria by two independent reviewers. This methodology ensured consistent and objective evaluations. Any discrepancies in the assessments were resolved through dialogue between the reviewers, and if necessary, the opinion of a third reviewer was sought. This ensured that the final selection of 10 articles accurately aligned with our specific objective in CCC, as shown in Table 2. Additionally, Figure 2 visually presented the geographical location of cases reported in the 10 selected articles from the Brazilian Amazon region.

**Table 2:** Selected articles in the literature review.

**References**	**Study type**	**Participants**	**Confirmed cases CCC**	**Origin**	**Positive serology**			
					**IFI**	**ELISA**	**Western-blot**	**IHA**
Viñas el at. (15)	Case report	2	2	Barcelos, Amazonas	2	2	2	
Salles et al. (16)	Case report	3	3	Barcelos, Amazonas	3	3	3	
Ferreira et al. (17)	Prospective cross-sectional	37	3	Manaus, Amazonas	3	2	3	
Ferreira et al. (17)	Case report	1	1	Manacapuru, Amazonas	1	1	1	
Ferreira et al. (17)	Case report	1	1	Cruzeiro do sul, Acre	1	1		
Andrade et al. (18)	Case report	1	1	Juruá, Amazonas	1	1	1	
Coura et al. (19)	Case-control	106	12	Barcelos, Amazonas	-	-	-	
Pinto el al. (20)	Cohort	179	5	Para and Amapa	1			1
Couceiro et al. (21)	Cross-sectional	1	1	Autazes, Amazonas	1	1	1	
Ortiz et al. (22)	Case report	53	2	Manaus, Amazonas	13	1	0	

ELISA, enzyme-linked immunosorbent assay; IFI, immunofluorescence; IHA, indirect hemagglutination assay.

To date, 25 cases of CCC have been reported in the Brazilian Amazon, and the clinical characteristics of these patients are summarized in Table 3.

**Table 3:** Electrocardiographic and echocardiographic abnormalities per patient.

**Case**	**Sex**	**Age**	**Echocardiogram**	**Ecocardiograma**		
				**Significant specific alterations**	**Apical aneurysm**	**LVFE**
1	M	45	AVB grade 1, LAA, LBBB, AVR	Dilated cardiomyopathy and pulmonary hypertension.		
2	F	44	VES, AVR	Dilated cardiomyopathy and pulmonary hypertension.		
3	M	53	RBBB + ASBB, VES, AVR.	Akinesia of the inferior and inferolateral walls, hypokinesia of the LV, dysfunction of the RV.	+	35%
4	M	69	RBBB + ASBB, VES.	Akinesia of the inferior and inferolateral walls, hypokinesia of the LV, dysfunction of the RV.	+	24%
5	M	70	LBBB, VES	Inferior akinesia.	+	29%
6	M	69	LVO	Diffuse hypokinesia.	+	28%
7	M	49	LBBB	Inferior akinesia.		30%
8	M	61	LVO	Dilated cardiomyopathy.		30%
9	M	48	AVB grade 2, VES	Hypokinesia of the inferior and posterior walls.	+	50%
10	M	49	RBBB	Dilated cardiomyopathy.	+	50%
11	M	60	AVR	Akinesia of the inferior wall.	+	28%
12	M	78	Normal	Diffuse hypokinesia.		26%
13	F	64	RBBB + ASBB	No alteration.		28%
14	F	20	RBBB, LBBB	No alteration.		71%
15	F	22	LBBB, AVR	Mild tricuspid regurgitation.		62%
16	M	14	Hemiblock of the posterior and inferior	Minimal increase in LV, decreased systolic performance, minimal mitral regurgitation.		69%
17	M	36	LAA, AVR	Left atrial overload, diastolic overload of the LV, dilated cardiomyopathy, mild mitral insufficiency.		45%
18	M	18	Sharp and asymmetric T wave, sinus bradycardia.	Dilated cardiomyopathy and pulmonary hypertension.		48%
^*^14-25			VES *n* = 12, Bradycardia *n* = 8, RBBB *n* = 4, LBBB *n* = 8, AVB *n* = 2, AVR *n* = 4			

ECG, electrocardiogram; LVEF, left ventricular ejection fraction; LVH, left ventricular hypertrophy; RBBB, right bundle bran; VES ch block, Ventricular extrasystoles; LBBB, left bundle branch block, ASBB, anterosuperior divisional block; VES, Ventricular extrasystoles; AVB, atrioventricular block; LAA, left atrial enlargement; AVR, Abnormal ventricular repolarization; LVO, Left ventricular overload; LAFB, left anterior fascicular block; PH, pulmonary hypertension; PE, pericardial effusion; LV, left ventricle.

^*^Cases 14–25 The article does not describe patients individually (19).

The first case report of CCC in the state of Amazonas was published in 2003. Two patients born and raised in the Rio Negro region of northern Amazonas presented with positive serology for *T. cruzi* and developed dilated cardiomyopathy and heart failure with reduced ejection fraction. Both patients had abnormal ECG findings, and they ultimately died from heart failure. This case report represents the first documented cases of CCC in the Amazon region (15).

In 2006, three new autochthonous CCC cases were reported from Rio Negro, Amazonas. All patients had heart failure symptoms. Patient 1 had RBBB with ASLDB, polymorphic ventricular premature beats, and ventricular repolarization changes on ECG. Patient 2 had RBBB with ASLDB and polymorphic ventricular premature beats. Both patients had left ventricular dilation, apical aneurysm, and impaired ventricular wall movement with severe global systolic dysfunction (LVEF 35% in patient 1, 24% in patient 2). Patient 3 had LBBB with isolated ventricular premature beats and similar echocardiographic findings as patients 1 and 2, along with severe global systolic dysfunction of the left ventricle (LVEF = 29%) and right ventricular dysfunction (16).

In 2009, the frequency of CCC was estimated in 37 autochthonous patients from the Amazon with left ventricular systolic dysfunction of undefined etiology. Three of the patients were diagnosed with CCC, with two cases showing left ventricular overload on ECG and one case with LBBB. Echocardiograms showed inferior akinesia, apical aneurysm, and left ventricular ejection fraction (LVEF) of 28% in the first patient, diffuse hypokinesia and LVEF of 30% in the second patient, and inferior akinesia and LVEF of 30% in the third patient. One of these cases was published with an image of a “glove-finger” type left ventricular apical aneurysm detected by magnetic resonance imaging (17).

In 2010, the first documented case of the predominantly thromboembolic form of CCC in the region was reported. The patient was diagnosed with an ischemic stroke and presented with second-degree AV block and ventricular ectopic beats on the ECG. Transthoracic echocardiography showed mild left ventricular systolic dysfunction with slight left ventricular enlargement, apical aneurysm, and an LVEF of 50%. The diagnosis of Chagas disease was confirmed by IFI and ELISA tests (23).

A case of aborted sudden death caused by ventricular tachycardia without a pulse was reported in 2012. The patient had RBBB on ECG, and the echocardiogram showed mild left ventricular enlargement, apical aneurysm, and hypokinesia of the inferior and inferolateral walls, with mild left ventricular systolic dysfunction. This was the first reported case of arrhythmogenic predominant form of CCC in the region (18).

In 2013, the first case-control study of Chagas cardiomyopathy in the region was conducted by Coura et al. The cases included 106 serologically positive patients for *T. cruzi* infection, who were matched with seronegative individuals. The electrocardiographic changes found in the cases were: ventricular extrasystoles (VE) in 12 (11.3%) patients, bradycardia in 8 (7.5%), RBBB in 8 (7.5%), LBBB in 4 (3.8%), AV block in 2 (1.9%), and ventricular repolarization abnormality in 4 (3.8%). The echocardiogram was abnormal in 22.6% of seropositive patients and 8.5% of seronegatives, with the main alterations found being septal hypokinesia, inferolateral wall akinesia, and a “glove-finger” apical aneurysm (19).

A follow-up study was conducted in 2013 on 179 individuals with CD from the states of Pará and Amapá in the Amazon region of Brazil. All of them were treated with benznidazole during the acute phase of the disease and were followed up for an average of 5.6 years. The study identified four clinical profiles based on paired analysis of serological, parasitological, cardiac, and digestive test results. Serological cure was observed in 26.3% of patients, while 73.7% showed persistence of anti-*T. cruzi* antibodies. Out of this group, 71% were characterized as indeterminate phase or seropositive depending on whether or not they had undergone digestive evaluation, and 2.8% had abnormalities consistent with the cardiac form of Chagas disease (20).

In 2021, researchers reported the successful implementation of a cardioverter-defibrillator in a patient with CCC. The patient had AVR in the lateral and inferior walls on the ECG, an apical aneurysm with an LVEF of 28% on echocardiogram, and significant left ventricular dysfunction revealed through magnetic resonance imaging, which also showed late transmural enhancement in the infero-medial-basal wall (21).

In 2021, 45 samples from patients with unexplained dilated cardiomyopathy in the Brazilian Amazon region were analyzed. Two serological tests, ELISA and indirect immunofluorescence, were performed, and samples with at least one positive serological reaction were subjected to molecular analysis. Two samples were positive and identified as TcIII/IV. In the first case, the ECG was normal, and echocardiography revealed akinesia in the infero-septal wall and an LVEF of 26%. In the second case, the ECG showed RBBB and ASLDB, and echocardiography revealed diffuse hypokinesia and an LVEF of 28% (22).

### Case series

#### Epidemiological data

The FMT-HVD has records of 131 patients with Chagas disease, including those in acute and chronic phases. This study describes the clinical characteristics of 25 patients with CCC attended between 2007 and 2022, of which 13 (52%) are from the state of Amazonas; 5 (20%) from Pará, 5 (20%) from Maranhão, and 2 (8%) from Acre. The median age was 51 years with an interquartile range (IQR) of 19 years, the mean age was 52.6 ± 11.4 years, ranging from 30 to 71 years, and 18 (72%) were male (Table 4). One patient in the study is from an outbreak of oral transmission by ingestion of contaminated palm fruit juice; in the others, the transmission route is unknown. During this period, 5 (20%) patients died, 2 (8%) due to heart failure, 1 (5%) due to sudden death, and 2 (8%) due to unknown causes. The most frequently reported symptom was dyspnea in 16 patients (64%), followed by palpitations in 9 (35%), precordial pain in 5 (20%), and dizziness in 5 (20%). All patients presented with cardiac alterations detected by at least one of the cardiac exams performed (Table 5). The main baseline characteristics of the patients are shown in Table 4.

**Table 4:** Characteristics of the study population.

	***Patients***	
	***%***	***N = 25***
**Gender**		
Female	28	7
Male	72	18
Age	51^*^	
**State/Region**		
Amazonas	52	13
Pará	20	5
Maranhão	20	5
Acre	8	2
**Transmission mechanism**		
Oral	4	1
Unknown	96	24
**Symptoms**		
Dyspnea	64	16
Chest pain	20	5
Palpitations	36	9
Dizziness	20	5
Syncope	12	3
Asthenia	8	2
Death	20	5

^*^Median age.

**Table 5:** Characteristics of the deceased cases.

	***Deaths***	
	***%***	***N =5***
**Sex**		
Female	20	1
Male	80	4
Age	54.2^*^	
**State/Region**		
Amazonas	60	3
Para	40	2
**Transmission mechanissm**		
Unknow	100	5
**Symptoms**		
Dyspnea	100	5
Dizzines	40	2
Syncope	20	1
Asthenia	20	1
**Cause of death**		
Heart failure	40	2
Sudden death	20	1
Unknow	40	2
***NYHA Class***		
C	40	2
D	60	3
**ECG**		
AVR	40	2
EIA	20	1
ASBB	60	3
LBBB	40	2
AVB 1	20	1
VES	20	1
RAO	20	1
LAO	20	1
LVO	20	1
**Echocardiogram**		
LVEF < 45%	100	5
LAI > 42 mL/m2	80	4
Segmental Alteration	100	5
Apical Aneurysm	40	2
LV Thrombus	20	1
**24-hour Holter monitor**		
Ventricular Extrasystole	100	5
Supraventricular Extrasystole	60	3
Non-sustained Ventricular Tachycardia	60	3
Pacemaker	20	1

^*^Median age.

VRA, Ventricular repolarization alterations; EIA, Electric inative area; RBBB, right bundle branch block; LBBB, left bundle branch block; ASBB, anterosuperior divisional block; AVB, atrioventricular block; CAVB, Complete atrioventricular block; AF, Atrial fibrillation; VES, Ventricular extrasystoles; RAO, Right atrial overload; LAO, Left atrial overload; LVO, Left ventricular overload; LVDD, left ventricular diastolic diameter; LVEF, left ventricular ejection fraction; LAI, indexed volume of the left atrium; LV, Left ventricle.

A Table 5 provides information on 5 recorded fatalities. 80% of the deceased are male, while 20% are female, with a mean age of 54.2 years. In terms of origin, 60% of the deceased are from Amazonas, and 40% are from Pará. Importantly, the transmission mechanism remains unidentified in all cases. Dyspnea emerges as the predominant symptom, present in 100% of the cases, followed by dizziness (40%), syncope (20%), and asthenia (20%). Regarding the cause of death, 40% is attributed to heart failure, 20% to sudden death, and 40% remain unknown. The New York Heart Association (NYHA) classification reveals that the majority of the fatalities fall into Class D (60%), followed by Class C (40%).

#### Cardiac alterations

Among the cohort studied, 80% of the individuals manifested discernible alterations in their electrocardiograms. The observed electrocardiographic variations encompassed: VRA in 12 individuals (48%), VES in 28%, ASBB in 28%, RBBB in 20%, LBBB in 16%, EIA in 12%, sinus bradycardia in 12%, AVB grade 1 in 8%, AVB grade 2 in 4%, CAVB in 4%, atrial fibrillation in 4%, right atrial overload in 4%, left atrial overload in 4%, and left ventricular overload in 4%.

Of the 25 echocardiograms performed, 80% (19) showed some type of alteration. The median left ventricular ejection fraction (LVEF) was 37, with an interquartile range (IQR) of 37 as well. 56% of individuals had an LVEF < 45%. The mean LVEF was 45.8 ± 4.04, ranging from 20 to 83. the median; the left ventricular diastolic diameter (LVDD) was >56 mm in 24% of individuals, with a mean of 59 ± 2.07 (range: 91–28), the median was 56, with an interquartile range (IQR) of 21; the indexed volume of the left atrium (LAI) was >34 mL/m^2^ in 40% of individuals, with a mean of 39.76 ± 1.49 (range: 58–26), the median was 40, with an interquartile range (IQR) of 13. Other alterations reported in the echocardiogram were: segmental alteration in 56% of patients, apical aneurysm in 44%, moderate mitral insufficiency in 12%, and moderate tricuspid insufficiency in 8%.

In the 24-h Holter monitor, we found that 18 (72%) patients had ventricular premature beats, 10 (40%) had supraventricular premature beats, 9 (36%) had unsustained supraventricular tachycardia, and 3 (12%) had paroxysmal supraventricular tachycardia (Table 6).

**Table 6:** Cardiological abnormalities per patient.

**Patient**	**Sex**	**Age**	**Electrocardiogram**	**Ecocardiograma**			**Abnormal Holter**	***NYHA***	***Death***
				**Significant specific alterations**	**Apical aneurysm**	**LVFE**			
1	M	57	ARV, EIA	Akinesia of the inferior and inferolateral walls, segmental alteration, left ventricular thrombus	+	25%	+	C	
2	M	31	Bradycardia, AVB	No alteration		69%		A	
3	F	65	LBBB	Segmental alteration	+	33%	+	D	
4	M	55	AVB2, VES	Segmental alteration	+	47%	+	B1	
5	M	37	LBBB, ASBB	No alteration		83%		B1	
6	M	39	LBBB	No alteration		73%		A	
7	F	58	RBBB. ASBB	No alteration		78%		A	
8	M	62	AVR, RBBB	Hypokinesia of the inferior wall	+	49%		B1	
9	F	52	AVR	Segmental alteration	+	65%		B1	
10	M	56	LBBB, AVB, AF	Hypokinesia of the inferior wall		20%	+	C	
11	M	30	VES	Diffuse hypokinesia		37%	+	D	
12	F	51	AVR, ASBB	Segmental alteration	+	60%	+	B1	
13	F	57	EIA, VES	Mild mitral insufficiency, akinesia septal, and akinesia of the inferolateral, anterolateral, and posterior walls	+	52%		D	
14	M	66	RBBB	No alteration		28%		D	
15	M	51	AVR, RBBB, VES	No alteration	+	35%		B2	
16	M	40	AVR, RBBB, ASBB	Akinesia septal	+	36%		D	
17	M	59	AVR, VES	No alteration		44%	+	B2	
18	F	40	AVR, VES	No alteration		68%		A	
19	M	71	AVR, RBBB, ASBB	Diffuse hypokinesia		77%	+	B1	
20	M	66	AVR	No alteration		41%		B2	
21	M	69	AVR, ASBB, RAO, VES	Akinesia of the inferior and inferolateral walls	+	28%	+	C	+
22	M	61	EIA, ASBB	Akinesia of the inferior and inferolateral walls		30%	+	D	+
23	M	45	LBBB, AVB1, LAO	Moderate tricuspid insufficiency, segmental alteration, left ventricular thrombus		22%	+	D	+
24	M	49	AVR, LBBB	Segmental alteration, diffuse hypokinesia		25%	+	D	+
25	F	47	ASBB, VES	Moderate mitral and tricuspid insufficiency, akinesia of the inferior, inferolateral, and anterior walls, pulmonary hypertension	+	22%	+	C	+

VRA, Ventricular repolarization alterations; EIA, Electric inative area; RBBB, right bundle branch block; LBBB, left bundle branch block; ASBB, anterosuperior divisional block; AVB, atrioventricular block; CAVB, Complete atrioventricular block; AF, Atrial fibrillation; VES, Ventricular extrasystoles; RAO, Right atrial overload; LAO, Left atrial overload; LVO, Left ventricular overload; LVDD, left ventricular diastolic diameter; LVEF, left ventricular ejection fraction; LAI, indexed volume of the left atrium; LV, Left ventricle.

The patients were classified based on the results of the ECG and echocardiography according to the classification of the Brazilian Society of Cardiology guidelines (7). The results are shown in Table 7.

**Table 7:** Classification of ECG and echocardiography results according to the guideline of the Brazilian Society of Cardiology.

	***Patients***	
	***%***	***N = 25***
A	16	4
B1	24	6
B2	12	3
C	16	4
D	35	8

By combining all the cases described in this review with all the cases described in the case series, we have the clinical characteristics of 55 patients with CCC from the Amazon region. Among these patients, the most common electrocardiographic abnormality was AVR, present in 40% of cases. In the echocardiogram, the most commonly found abnormality was left ventricular systolic dysfunction (49%), followed by akinesia or hypokinesia of the inferior and/or inferolateral walls (38.1%), and the presence of apical aneurysm (32.7%). The description of these 55 associated cases is shown in Table 8.

**Table 8:** Electrocardiographic and echocardiographic alterations in patients with CCC autochthonous to the Amazon.

	***N** = **55***	
	***Patients***	
	***%***	***N = 55***
**ECG**		
AVB	14.5	8
RAO	23.6	13
LBBB	30.9	17
AVR	40	22
VES	34.5	19
RBBB	25	14
ASBB	18.1	10
LVO	5.4	3
EIA	5.4	3
Sinus bradycardia	21.8	12
AF	1.8	1
LAO	1.8	1
Inferior-posterior hemiblock	1.8	1
Asymmetric peaked T wave	1.8	1
**Echocardiogram**		
Systolic dysfunction of the left ventricle	49	27
Systolic dysfunction of the right ventricle	3.6	2
Inferior and/or inferolateral segmental alteration	38.1	21
Apical aneurysm	32.7	18
Thrombus in the left ventricle	5.4	3
Pulmonary hypertension	3.6	2
Significant valve disease	9	5

AVB, Atrioventricular Block; RAO, Right atrial overload; LBBB, Left bundle branch block; VRA, Ventricular repolarization alterations; VES, Ventricular extrasystoles; EIA, Electric inative area; RBBB, Right bundle branch block; ASBB, Anterosuperior divisional block; LVO, Left ventricular overload; AF, Atrial fibrillation; LAO, Left atrial overload.

## Discussion

This work constitutes a thorough documentation of all reported cases of chronic Chagas heart disease (CCC) in the Brazilian Amazon region. Initially, an exhaustive review of all published articles and documents concerning CCC in the Amazon was conducted. Additionally, a case series of patients with CCC, being cared for and monitored by specialized cardiologists in an outpatient setting exclusively dedicated to Chagas disease, was presented. This meticulous approach enabled a comprehensive collection of clinical, historical, and laboratory data, bridging a significant information gap commonly found in previous studies.

We have identified a significant gap between reported cases and the high prevalence of the acute phase. This difference may be partly attributed to potential detection biases and inherent challenges in ascertainment within the epidemiological context of Chagas disease in this particular geographic area.

One of the potential biases stems from the different transmission routes, particularly the oral route prevalent in the Amazon region. Symptoms associated with the oral transmission of Chagas disease tend to be more evident, leading affected individuals to seek timely medical attention. Consequently, there may be an overrepresentation of acute cases in the reported data due to heightened awareness and proactive healthcare-seeking behavior among symptomatic individuals. Rapid diagnosis and subsequent early administration of treatment, using benznidazole (Rochagan^®^) (3), could alter the natural course of the disease, potentially distorting the representation of disease progression in the Amazon region (24).

Furthermore, another significant potential bias in Chagas disease detection in the Amazon region arises from the population seeking cardiology services, often representing individuals in an advanced and symptomatic stage of the disease. These patients seek cardiac care due to evident cardiac manifestations, indicating an advanced state of cardiac damage. This biased representation in healthcare-seeking behavior may underestimate the true burden of CCD in the region, further underscoring the need to enhance detection mechanisms during the earlier states of the chronic phase (17).

In the Amazon region, commercial tests showed significantly reduced effectiveness compared to other parts of Brazil (25). In their 2007 publication, Aguilar et al. highlighted a high propensity for false positives in conventional serological tests in the Amazon region (26). In contrast, Ortiz et al. (22) published work, questioned whether the low prevalence of CCC could actually be due to underdiagnosis. In this cross-sectional study, patients with a cardiac profile indicative of idiopathic dilated cardiomyopathy were recruited. A total of 45 samples were analyzed using ELISA and immunofluorescence, without confirming CCC cases. It is important to note that the test used in this study demonstrated the best performance for this region (25). The samples underwent molecular analysis, identifying two as TcIII/IV. However, the presence of *T. cruzi* DNA does not necessarily confirm CCC, suggesting possible genotypic coincidences in patients with ventricular dysfunction but without a positive serological diagnosis. Although the study was limited in size, it confirms the issue of low reactivity in commercial serological kits, as well as the potential cross-reactivity with endemic diseases. These discrepancies in the scientific literature underscore the ongoing need for clinical and epidemiological research to determine the true situation of CCC in the Amazon region.

It is pertinent to highlight that a crucial step was taken in 2020 with the designation of chronic Chagas disease (CCD) as a notifiable disease at the national level in Brazil. This strategic measure holds great potential in addressing these potential detection biases by promoting standardized reporting, enhancing medical care for affected individuals, and facilitating coordinated surveillance efforts to capture a more accurate representation of chronic cases. Strengthening healthcare infrastructure, increasing awareness, and optimizing diagnostic procedures, especially for chronic cases, are imperative to comprehensively understand the true burden and progression of CCD in the Amazon region (10).

Different from other regions in Brazil where there are more cases of CCC, the DTUs in our region are mainly TcI and TcIV (27, 28). Integrating findings from a study on *T. cruzi* tropism in *Octodon degus*, a naturally infected rodent, a predominance of TcV DTU was identified in key organs such as the esophagus, skin, skeletal muscle, brain, and intestines (29). This indicates an extensive tropism of the parasite in chronically infected O. degus. TcV, commonly associated with human infections in the Southern Cone of South America, was the prevalent strain in over 90% of the analyzed organs. Considering these results in the context of the region under study, where a predominance of TcIV strains has been observed in chronic Chagas disease patients, an intriguing speculation arises. There might be a correlation between the prevalent DTU (TcIV) in this region and a less pronounced tropism of the parasite toward critical organs for the development of CCC, such as the heart. Based on the observed reduced tropism in O. degus with the TcV strain, we can infer that TcIV, being prevalent in this region, could be associated with a lesser progression of cases toward cardiac disease in human patients (30).

Right bundle branch block (RBBB), whether complete or incomplete, is the most common conduction disorder observed in Chagas disease, affecting 10% to 50% of infected patients and often coexisting with anterosuperior divisional block (ASDB) (7). In our population, RBBB was found in 24% of cases, indicating a relatively high prevalence. Notably, the presence of left bundle branch block (LBBB) was observed in 30% of cases, which is a rare and late manifestation of chronic Chagas disease reported in other regions and associated with poor prognosis for affected individuals (7). These findings underscore the importance of close monitoring and timely interventions to prevent adverse cardiac outcomes in Chagas disease patients, particularly those with conduction disorders.

In a study conducted in 2007, it was found that approximately half of symptomatic Chagas disease patients present with apical aneurysms on echocardiography (31).

Our study found a similar prevalence of apical aneurysms in the Amazon region. Apical left ventricular aneurysms can be useful for the etiological diagnosis of dilated cardiomyopathy.

Additionally, we observed hypokinesia or akinesia of the inferior and inferolateral walls in 55% of cases. These findings, along with increased diastolic dimensions of the left ventricle, are frequently reported in advanced stages of Chagas cardiomyopathy (31)The identification of these echocardiographic abnormalities can aid in the diagnosis and management of Chagas disease patients, especially those with advanced stages of the disease.

In 2022, Couceiro et al. described myocardial lesions through cardiovascular magnetic resonance imaging in a group of patients with acute Chagas disease in the Amazonian region. Myocardial fibrosis was observed in 58% of cases in the pre-treatment phase and in 70% of cases 1 year after treatment completion (31). During the analysis of affected segments in the acute phase, it was found that 16% showed late gadolinium enhancement in the anteroseptal wall, another 16% in the inferoseptal wall, and 25% in the inferolateral wall. Notably, a relatively low frequency of alterations was observed in the inferolateral wall, a region classically implicated in Chagas disease in endemic areas (32).

These acute myocardial findings, especially the involvement of the anteroseptal wall, can significantly influence subsequent cardiac conduction. Considering the progression from acute findings to evolving stages, it becomes evident how these myocardial alterations, particularly in the anteroseptal region, could explain the higher frequency of left bundle branch block (LBBB) observed in the Amazonian region. This correlation underscores the potential impact of acute myocardial involvement on subsequent electrocardiographic manifestations like LBBB. This becomes even more apparent when merged with the established knowledge of the persistence of fibrosis and myocardial inflammation across all chronic phases of Chagas disease.

Furthermore, consistent demonstration of fibrosis and myocardial inflammation has been observed throughout all chronic phases of Chagas disease, with the amount of fibrosis correlating with the severity of the clinical form. The identified alterations during the acute phase may serve as predictive indicators for future bundle branch block development, shedding light on the distinctive patient profile with CCC in the Amazonian region. Additionally, diagnostic challenges prevalent in the region may lead to delayed CCC diagnosis, potentially explaining the heightened prevalence of RBBB in the Amazonian region.

Our study emphasizes the critical importance of increasing awareness of CCC among healthcare professionals in the Amazon region. Early detection of CCC proves to be a fundamental factor in improving the prognosis and quality of life for affected patients. Although our research suggests a potentially lower incidence of CCC compared to other regions, the cases exhibit comparable clinical characteristics and severity.

It is imperative to implement effective strategies for early identification of CCC, especially during the acute phase, where a high frequency of anterior and septal involvement, as well as right bundle branch block, is evident. This approach would enable more timely and effective treatment, reducing the progression to serious complications associated with the disease, such as cardiac arrhythmias and heart failure.

Additionally, we highlight the pressing need to promote ongoing education and updates for healthcare professionals in the region regarding the clinical aspects and peculiarities of CCC. This initiative will contribute to a more precise identification of cases and early intervention, ultimately improving outcomes and the quality of life for patients affected by this disease in the Brazilian Amazon region. These conclusions urge immediate action to address CCC more effectively and enhance patient care in the region.

## Data availability statement

The original contributions presented in the study are included in the article/supplementary material, further inquiries can be directed to the corresponding author.

## Ethics statement

The studies involving humans were approved by the FMT-HVD's Research Ethics Committee approved this study under the CAAE number 47017121.6.0000.0005. The studies were conducted in accordance with the local legislation and institutional requirements. The participants provided their written informed consent to participate in this study. Written informed consent was obtained from the individual(s) for the publication of any potentially identifiable images or data included in this article.

## Author contributions

EG: Conceptualization, Data curation, Investigation, Methodology, Resources, Visualization, Writing—original draft. SS: Investigation, Resources, Writing—review & editing. JO: Resources, Writing—review & editing. DT: Investigation, Writing—review & editing. VM: Investigation, Writing—review & editing. KN: Investigation, Writing—review & editing. AB: Investigation, Writing—review & editing. JG: Supervision, Validation, Visualization, Writing—review & editing. MV: Supervision, Validation, Visualization, Writing—review & editing. JB: Supervision, Writing—review & editing, Conceptualization, Resources, Visualization.
